# A stable enol from a 6-substituted benzanthrone and its unexpected behaviour under acidic conditions

**DOI:** 10.3762/bjoc.5.31

**Published:** 2009-06-16

**Authors:** Marc Debeaux, Kai Brandhorst, Peter G Jones, Henning Hopf, Jörg Grunenberg, Wolfgang Kowalsky, Hans-Hermann Johannes

**Affiliations:** 1 Labor für Elektrooptik am Institut für Hochfrequenztechnik, Technische Universität Braunschweig, Bienroder Weg 94, 38106 Braunschweig, Germany; 2 Institut für Organische Chemie, Technische Universität Braunschweig, Hagenring 30, 38106 Braunschweig, Germany; 3 Institut für Anorganische und Analytische Chemie, Technische Universität Braunschweig, Hagenring 30, 38106 Braunschweig, Germany

**Keywords:** consecutive reactions, intramolecular cyclization, molecular modelling, spiro compounds, X-ray structural analysis

## Abstract

Treatment of benzanthrone (**1**) with biphenyl-2-yl lithium leads to the surprisingly stable enol **4**, which is converted by dehydrogenation into the benzanthrone derivative **7**. Under acidic conditions **4** isomerises to the spiro compound **11** and the bicyclo[4.3.1]decane derivative **12**. Furthermore, the formation of **7** and the hydrogenated compound **13** is observed. A mechanism for the formation of the reaction products is proposed and supported by DFT calculations.

## Introduction

Compounds for optoelectronic applications with electroluminescent (e.g. organic light-emitting diodes, OLEDs) or light-harvesting properties (e.g. organic solar cells) are receiving more and more attention [1]. In this respect benzanthrone (**1**), with its luminescent and photosensitizing properties, is an interesting candidate for the construction of these systems. Recently, aminobenzanthrone derivatives have been shown to be efficient emitters for OLED applications [2]. In these devices, the benzanthrone moiety acts as an electron accepting group, whereas the diarylamine group functions as an electron donor.

**Scheme 1:**
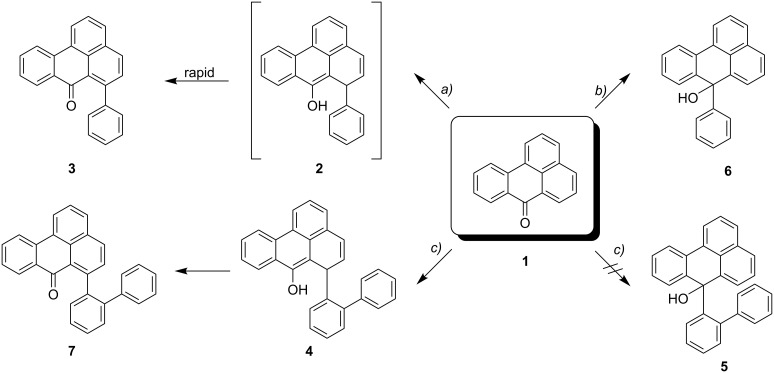
Behaviour of benzanthrone (**1**) towards phenylmagnesium chloride (*a*), phenyl lithium (*b*), and biphenyl-2-yl lithium (*c*).

The reaction of **1** with various organometallic reagents was studied by Allen in the 1970s [3]. It was shown that an attack of phenylmagnesium chloride or phenyl sodium after 1,4-addition leads to the 6-substituted benzanthrone derivative **3** (Scheme 1). On changing the solvent from ether-benzene to tetrahydrofuran the ketone could also be isolated in high yields, but additionally a labile enol was produced that was hard to separate. To this compound, obviously an intermediate in the addition process, the authors assigned structure **2**, a compound that under the reaction condition is dehydrogenated to **3**.

Here, we present the first isolable enol derived from a benzanthrone and the unexpected behaviour of this adduct under acidic conditions.

## Results and Discussion

### Syntheses

Benzanthrone (**1**) was treated with biphenyl-2-yl lithium (Scheme 1). After work-up and chromatography the surprisingly stable enol **4** was obtained in 56% yield. However, no formation of the tertiary alcohol **5** could be observed, a compound type which is produced (derivative **6**) when benzanthrone was treated with phenyl lithium [3]. The yield of **4** was not improved by addition of a copper(I) salt in catalytic amounts [4]. This procedure should have favoured the ratio of a 1,4- to a 1,2-addition product [5].

**Figure 1:**
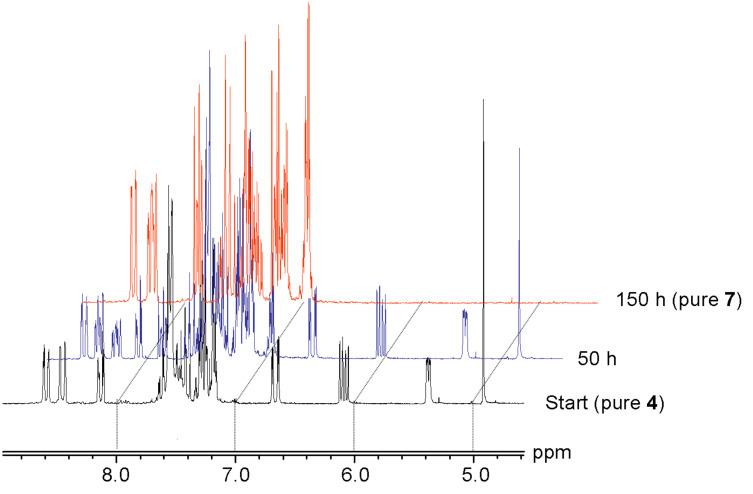
^1^H NMR spectra (200 MHz) of **4** in CDCl_3_ solution and time dependence.

**Scheme 2:**
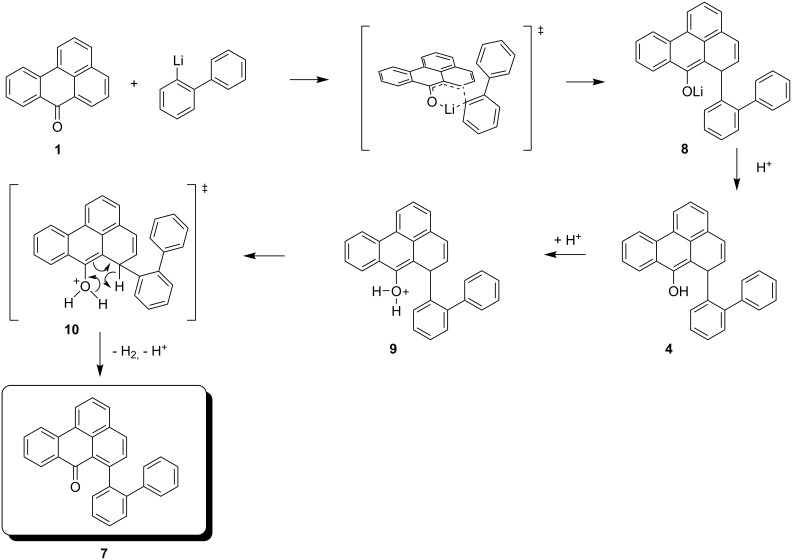
Proposed mechanism for the formation of **4** and its oxidation to **7**.

The enol **4** is stable as a solid and also in deuterated dimethyl sulfoxide, since an NMR solution in this solvent was unchanged after one week. In contrast, a solution of **4** in chloroform showed quantitative conversion to the 6-substituted benzanthrone **7** after approximately one week; a process that was subsequently monitored by ^1^H NMR spectroscopy (Figure 1) in CDCl_3_. This conversion is much slower when the CDCl_3_ is filtered through an alumina plug before use. The reaction constitutes a formal dehydrogenation of **4** (Scheme 2).

**Scheme 3:**
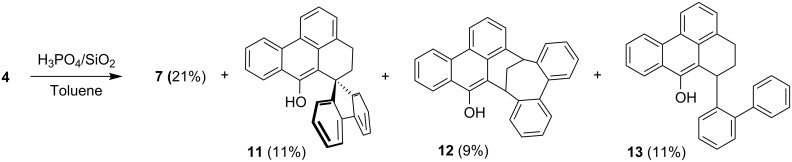
Conversion of the enol **4** under acidic conditions and reaction products.

As shown in Scheme 2, we propose that the formation of **4** and **7** starts as a 1,4-addition process as discussed above via the enolate **8** as an intermediate. From this, the enol **4** is generated under the influence of the added acid. Further protonation provides the oxonium ion **9** which is set up for a retro-[2+4]-cycloaddition (see transition state **10**) to lose hydrogen and finally become deprotonated to yield the isolated **7**. Since at this stage of our study we were not interested in mechanistic investigations we did not look for the production of hydrogen. Considering the small amount of substrate we were working with (0.7 mM concn of **4**) and the slow process of the conversion, it is not surprising that we could not see any gas formation (hydrogen bubbles). However, what makes this rationalisation attractive is the production of both an aromatic system as well as a carbonyl group, so the process is thermodynamically favourable. Furthermore, the formation of quinomethides from ortho-substituted phenols is a well known phenomenon in mass spectrometry (the “ortho-effect” see [6]). Next, the enol **4** was treated deliberately under acidic conditions by heating it with phosphoric acid in toluene under reflux. Silica gel was added to the two-phase mixture in order to effect a better contact between the layers. The progress of the reaction was monitored by TLC, which indicated that, surprisingly, three different new compounds were produced besides **7** (21% yield). After work-up and chromatography, these new products were identified as **11**, **12**, and **13** (Scheme 3). The ketone **7** itself is stable under these reaction conditions.

Spiro compound **11** (11% yield) was characterised by NMR spectroscopy, mass spectrometry and single crystal X-ray crystallography (see below). The ^1^H NMR spectrum (600 MHz) of **11** shows two aliphatic triplets at δ = 2.16 and 3.42 ppm (*J* = 6.2 Hz) which are assigned to the four methylene protons. In the ^13^C NMR spectrum (151 MHz) the corresponding carbon atoms cause signals at 28.4 and 37.3 ppm, respectively. The spiro carbon atom is represented by a singlet at 53.4 ppm. All other spectroscopic data correspond to expectations and are recorded in the Experimental section.

Compound **12** was characterised by NMR spectroscopy and mass spectrometry. The proton signals at δ = 2.61 and 2.84 ppm (400 MHz) with a geminal coupling constant of 13.5 Hz correspond to the protons of the methylene bridge. The bridgehead protons arise as a multiplet at 4.70–4.76 ppm. NMR data of the aliphatic protons correlate well with the data for 1,6-dihydro-1,6-methanobenzo[*d*]cyclooctene, a compound with a similar carbon framework described by Banciu and co-workers [7].

Compound **13** was characterised by NMR spectroscopy, mass spectrometry and by single crystal X-ray crystallography (see below). The mass spectrum of compound **13** shows a signal with *m/z* = 386, which exceeds the molar mass of the starting material **4** by 2 Da. The ethylene moiety is represented in two groups of multiplets in the ^1^H NMR spectrum (400 MHz; δ = 1.68–1.77 and 2.79–2.84 ppm).

**Scheme 4:**
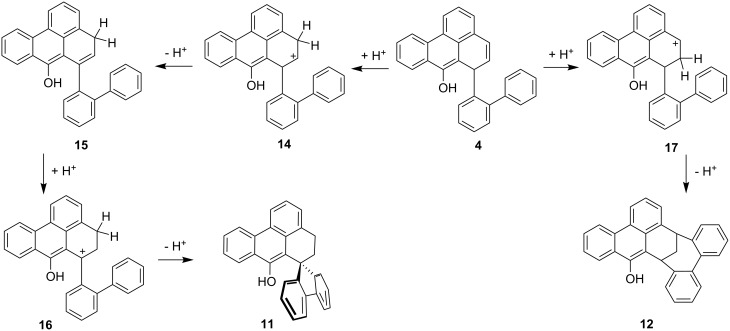
Proposed mechanism for the formation of spiro compound **11** and bicyclo[4.3.1]decane derivative **12**.

The formation of these three new compounds can be explained as follows. For the production of **11** and **12** we propose the mechanism summarised in Scheme 4 [8].

Both **11** and **12** have the same molecular mass as the starting material **4**, so the processes leading to these two products are isomerisations. The protonation that initiates the rearrangements can take place at C-4 or C-5 of the starting material **4**. In the former case the secondary cation **14** results, which by proton loss is converted into hydrocarbon **15**; in other words, **4** has undergone an acid-catalyzed allylic rearrangement. Renewed protonation leads to the tertiary cation **16**, which by an internal Friedel-Crafts alkylation provides the spiro compound **11**. Alternatively, protonation of **4** at C-5 generates the benzylic cation **17**, which by intramolecular electrophilic attack leads to the bicyclo[4.3.1]decane derivative **12**.

**Scheme 5:**
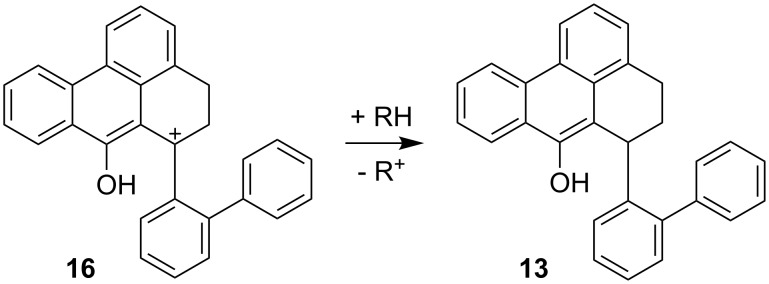
Proposed mechanism for the formation of **13**.

Finally, the formation of **13** is a formal hydrogenation of the starting material **4**. In the absence of a catalytically active layer that promotes a hydrogen-transfer reduction [9–10], we propose an acid-catalysed hydride transfer of the type reported by Carlson and Hill [11]. Thereby, a carbenium ion such as **14**, **16** or **17** (only the case of **16** is discussed in the following) can abstract hydride from another molecule that itself forms a stable cation (Scheme 5).

In order to make the above mechanistic speculations more than simple “electron pushing”, we decided to apply the following computational methods.

### Reaction mechanisms by computational methods

The gas phase global minima of the relevant molecules **4**, **7** and **9**–**18** were obtained by first applying an extended conformational analysis using the OPLS2005 force field [12] together with a Monte Carlo torsional sampling as implemented in the Macromodel 9.5 program [13]. Each lowest energy conformation of **4**, **7** and **9**–**18**, respectively, was then optimised by applying density functional theory. The M05-2X hybrid functional [14] was employed, and all atoms were described by a standard triple zeta all electron basis set augmented with one set of polarization functions (6-311G(d,p)). After the relevant stationary points were localised on the energy surface, they were further characterised as minima states by normal mode analysis based on the analytical energy second derivatives. Enthalpic and entropic contributions were estimated from the partition functions calculated at room temperature (298 K) under a pressure of 1 atm using Boltzmann thermostatistics and the rigid rotor harmonic oscillator approximation as implemented in the Gaussian03 set of programs [15]. Table 1 summarises the reaction energies/enthalpies of the different reaction steps. Although all calculations were carried out in the gas phase, it can be assumed that solvation effects will not counterbalance such high energetic differences.

**Table 1:** Gas phase electronic energies/enthalpies for intermediates generated along the proposed reaction pathway. All values are given in kcal mol^−1^.

	Δ*E*_0_	Δ*H*_298_	Δ*G*_298_

**4** + H^+^ → **9**	−193.95	−193.82	−193.96
**9** → **7** + H_2_ + H^+^	204.64	206.37	196.59

**4** → **7** + H_2_	**10.69**	**12.56**	**2.62**

**4** + H^+^ → **14**	−194.38	−194.52	−194.32
**14** → **15** + H^+^	194.29	194.45	194.17
**15** + H^+^ → **16**	−223.59	−223.35	−224.97
**16** → **11** + H^+^	206.49	205.75	208.77

**4** → **11**	**−17.19**	**−17.68**	**−16.34**

**4** + H^+^ → **17**	−212.87	−212.63	−214.39
**17** → **12** + H^+^	206.32	205.19	209.39

**4** → **12**	**−6.55**	**−7.45**	**−5.00**

**4** + H^+^ → **14**	−194.38	−194.52	−194.32
**14** → **15** + H^+^	194.29	194.45	194.17
**15** + H^+^ → **16**	−223.59	−223.35	−224.97
**16** + H^−^ → **13**	−207.23	−208.68	−200.75
H_2_ → H^+^ + H^−^	407.16	406.57	408.09
**4** → **18** + H^−^	187.61	188.95	180.77
**18** → **7** + H^+^	230.24	230.17	229.94

**4** + H_2_ → **13**	**−23.75**	**−25.54**	**−17.78**

The proposed reaction pathway from **4** to **7** proceeds via a protonation of the OH group to form **9**, releasing 193.95 kcal mol^−1^. This may trigger a H_2_ abstraction in a concerted manner, followed by a proton abstraction, which demands 204.64 kcal mol^−1^. The net transformation **4** → **7** + H_2_ is slightly endothermal (10.69 kcal mol^−1^), thus the driving force of the ketone formation is the generation of dihydrogen.

The protonation of **4** at C-4 results in the secondary ion **14** which releases 194.38 kcal mol^−1^. The proton loss of **14** to form **15** costs 194.29 kcal mol^−1^. Further protonation of **15** (−223.59 kcal mol^−1^) generates the cation **16**, which can easily undergo an internal electrophilic substitution to form the spiro compound **11** (206.49 kcal mol^−1^). This results in an overall exothermicity of 17.19 kcal mol^−1^ for the reaction **4** → **11**.

In order to explain the formation of the bicyclo[4.3.1]decane derivative **12** the enol **4** is protonated first at C-5 which releases 212.87 kcal mol^−1^. The benzyl cation thus generated can undergo a similar electrophilic substitution to produce the final product **12** at an effort of 206.32 kcal mol^−1^, resulting in an overall exothermicity of −6.55 kcal mol^−1^ for the reaction **4** → **12**.

**Scheme 6:**
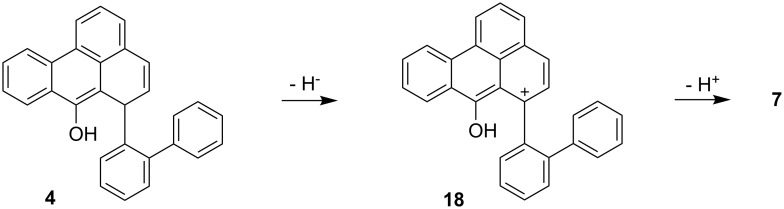
Proposed mechanism for the formation of **18** as a hydride source and further conversion to **7**.

The formation of **13** can be explained by addition of a hydride species. Although the reaction takes place under acidic conditions, the reaction of **4** to **18** for example (Scheme 6) can deliver H^−^ at a cost of 187.61 kcal mol^−1^ while the addition of a hydride to **16** releases 207.23 kcal mol^−1^. The cation **18** can then undergo a proton abstraction to form the ketone **7** (230.24 kcal mol^−1^). The reaction **4** → **7** + H_2_ and **4** + H_2_ → **13** can thus be seen as essentially coupled and in sum exothermic (−13.06 kcal mol^−1^).

### X-Ray structural analyses

**Figure 2:**
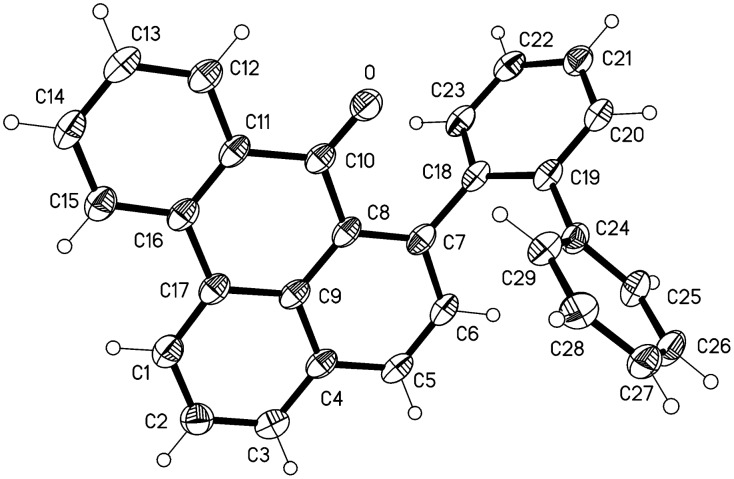
Ellipsoid representation (50% level) of compound **7** in the crystal.

**Figure 3:**
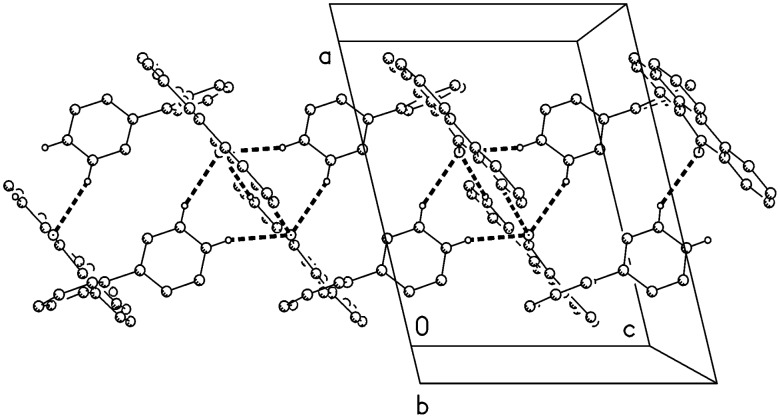
Packing diagram of compound **7** viewed parallel to **b**; hydrogen bonds C-H···O are indicated by dashed lines.

The molecule of compound **7** is shown in Figure 2. Bond lengths and angles may be regarded as normal; the bond length C10-O of 1.223(2) Å clearly indicates a double bond. The main ring system C1-C17 is planar (rmsd 0.040 Å), whereby the O atom lies 0.27 Å out of the plane and the ring C18-23 subtends an interplanar angle of 67.5°. The packing is mainly characterised by the weak C-H···O interactions from H12, H27 and H28, each with H···O 2.60 Å, forming via inversion and *z* translation operators a chain of molecules parallel to **c** (Figure 3).

**Figure 4:**
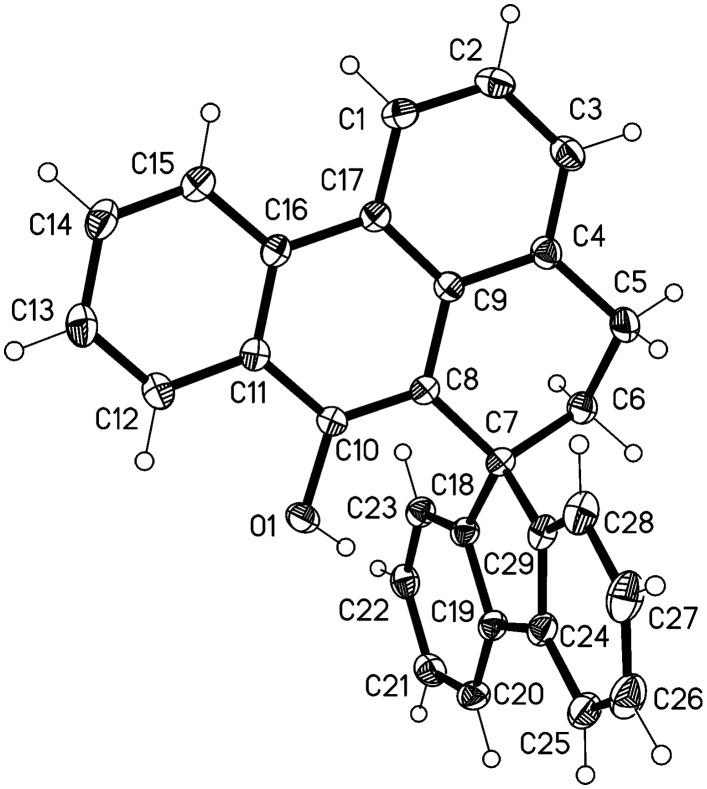
Ellipsoid representation (50% level) of compound **11** in the crystal.

**Figure 5:**
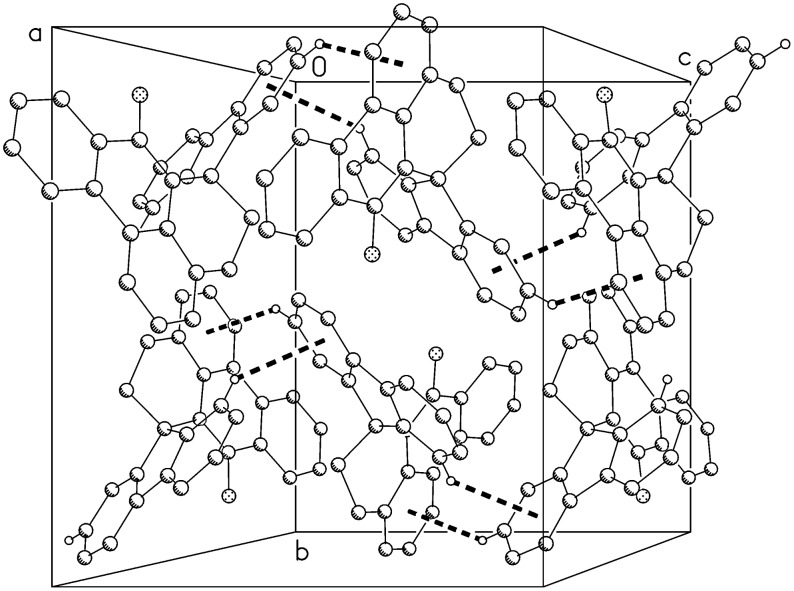
Packing diagram of compound **11** viewed perpendicular to the **bc** plane; hydrogen bonds C-H···π are indicated by dashed lines.

The molecule of compound **11** is shown in Figure 4. The bond length C10-O1 of 1.375(1) Å is consistent with a single bond, and the hydroxy hydrogen atom was located and freely refined. The main ring system (including the oxygen atom, but excluding C5 and C6) is reasonably planar, with a rmsd of 0.074 Å; the rings C18-23 and C19-24 are essentially coplanar (interplanar angle 3.9°). Despite the presence of the hydroxyl group, there are no classical hydrogen bonds; instead, the OH hydrogen is directed towards the ring centroid of C18-23 (H···centroid 2.74 Å, angle 163°). The packing involves two short H···π contacts, H22···centroid (C1-4,9,17) 2.56 Å and H28···centroid (C18-23) 2.74 Å, both via the glide operator, resulting in chains of molecules parallel to **c** (Figure 5).

**Figure 6:**
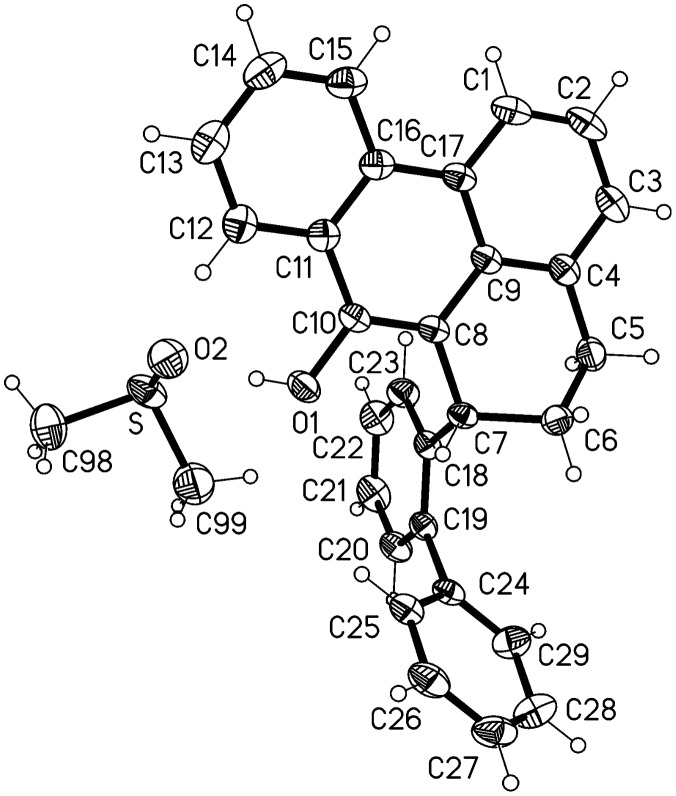
Ellipsoid representation (50% level) of compound **13** (d_6_-DMSO solvate) in the crystal. Hydrogen bonds (see text) are not drawn explicitly.

**Figure 7:**
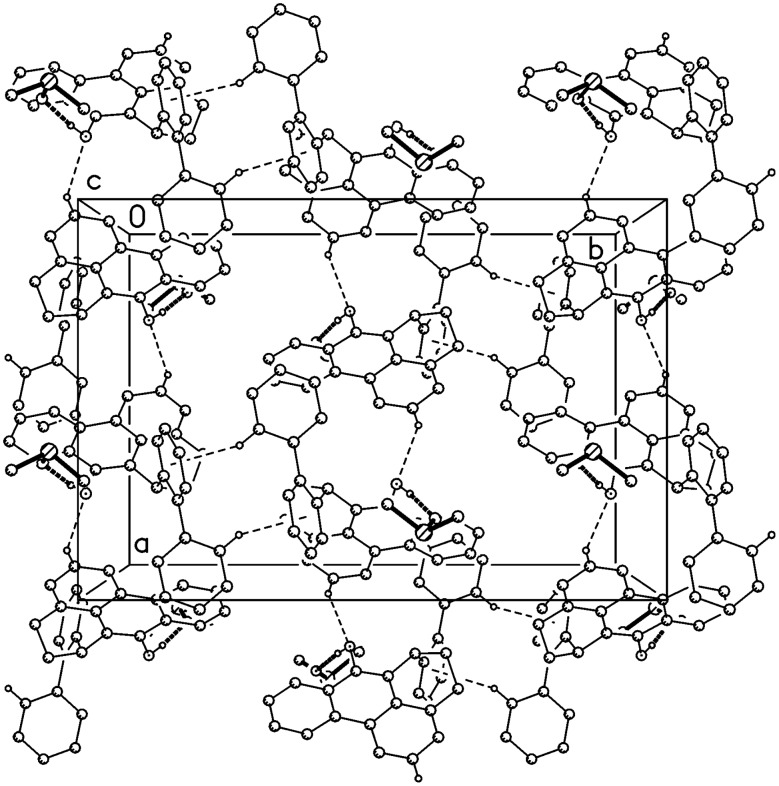
Packing diagram of compound **13** viewed parallel to **c**; DMSO molecules (including their hydrogen bonds) are represented by thick bonds. Hydrogen bonds C-H···O and C-H···π are indicated by thin dashed lines. The interactions D99A···π (see text) are omitted for clarity, but their positions are implicit (on the opposite side of the ring from the C-H···π interaction already drawn).

Compound **13** crystallizes with one molecule of deuterated DMSO; the formula unit is shown in Figure 6. The DMSO is well-ordered and is involved in a classical hydrogen bond O1-H01···O2 from the hydroxyl group, with H···O 0.87(2), O···O 2.655(1) Å, O-H···O 174(2)°. The DMSO methyl deuterium D99A forms a short H···π contact of 2.54 Å to the centre of the ring C18-23. The bond length C10-O1 of 1.369(1) Å is consistent with a single bond, and the hydroxy hydrogen was located and freely refined. The ring system C1-C17, less C6, is planar to within an rmsd. of 0.055 Å, and the ring C18-23 subtends an angle of 88.4(1)° with the plane so defined; the rings C18-23 and C24-29 subtend an angle of 64.0(1)°. The extended packing (Figure 7) involves the hydrogen bonds H2···O1 2.61 Å and H29···π(C18-23) 2.55 Å; the overall effect is to form strongly corrugated layers perpendicular to the *z* axis.

## Experimental

### General

Melting points: Stuart Melting Point SMP3 apparatus, uncorr. – Elemental analyses: Vario EL Elemental Analysis Instrument (Elementar Co.). – IR: Bruker Tensor 27 spectrometer with a Diamond ATR sampling element. – UV/Vis: Varian Cary 100 Bio as solutions in spectroscopic grade solvents. – NMR: Bruker DPX-400 and AV2-600 spectrometers; ^1^H chemical shifts were recorded relative to tetramethylsilane (TMS) as internal standard and ^13^C measurements are referred to the corresponding NMR solvent signal. All *J* values are in Hertz and are rounded to the nearest 0.1 Hz. – MS: Thermofinnigan MAT95. – TLC: SiO_2_ plates (Polygram SIL G/UV 254). – All compounds were purified by flash chromatography on Kieselgel 60 (Fluka). All reagents, unless otherwise specified, were obtained from Aldrich, Acros and Fluka and used as received. All solvents were purified before use. All reactions were performed under nitrogen atmosphere. Dry solvents stored over molecular sieve were purchased from Fluka.

#### 6-(Biphenyl-2-yl)-6*H*-benzo[*de*]anthracen-7-ol (**4**)

To a solution of 2-bromobiphenyl (2.00 g, 8.58 mmol) in dry THF (20 mL) a 1.6 M solution of *n*-butyl lithium in *n*-hexane (6.4 mL, 10.3 mmol) was added drop wise at −80 °C. After 1 h of stirring at the same temperature, the solution was added to a suspension of benzanthrone (**1**) (1.98 g, 8.58 mmol) in dry THF (20 mL). The brownish mixture was stirred for 2.5 h under reflux before allowing to cool to room temperature. A further 16 h of stirring was followed by quenching with a saturated aqueous solution of ammonium chloride (200 mL). After extraction with CHCl_3_ (3 × 100 mL) the combined organic phases were dried (MgSO_4_) and the solvents were evaporated. The brown crude product was purified by flash chromatography (CH_2_Cl_2_/*n*-hexane, 1:1, v/v; *R*_f_ = 0.66) to give 1.89 g (56%) of the enol **4** as a light-yellow solid with mp 235 °C. IR: 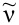  = 3555 (w), 3063 (w), 3021 (w), 1593 (w), 1493 (w), 1473 (w), 1457 (w), 1410 (w), 1331 (w), 1270 (w), 1202 (m), 1157 (w), 1096 (w), 1042 (w), 1007 (w), 982 (w), 922 (w), 896 (w), 836 (w), 821 (m), 742 (vs), 705 (s) cm^−1^. UV (acetonitrile): λ_max_ (log ε) = 204 (4.82), 229 (4.66), 256 (4.39), 268 (4.44), 286 (4.31), 319 (3.92), 332 (4.05), 347 (3.97), 378 (3.33) nm. ^1^H NMR (400 MHz, d_6_−DMSO): δ = 5.56 (br. d, *J* = 4.7 Hz, 1 H), 5.95 (dd, *J* = 9.6, 4.8 Hz, 1 H), 6.60 (dd, *J* = 9.7, 1.6 Hz, 1 H), 6.75 (br. d, *J* = 7.9 Hz, 1 H), 7.05 (ddd, *J* = 7.9, 6.8, 2.1 Hz, 1 H), 7.13–7.20 (m, 2 H), 7.31 (br. d, *J* = 6.9 Hz, 1 H), 7.40–7.47 (m, 2 H), 7.56 (br. dd, *J* = 7.5, 7.5 Hz, 2 H), 7.60–7.72 (m, 4 H), 8.23–8.26 (m, 1 H), 8.59 (br. d, *J* = 8.7 Hz, 1 H), 8.80 (dd, *J* = 7.7, 1.9 Hz, 1 H), 9.39 ppm (s, 1 H, OH). ^13^C NMR (101 MHz, d_6_−DMSO): δ = 117.3 (s), 122.0 (d), 122.3 (d), 123.2 (d), 124.1 (d), 124.5 (d), 124.7 (d), 124.9 (s), 125.7 (d), 126.0 (s), 126.3 (d), 126.4 (d), 127.0 (d), 127.3 (d), 127.7 (d), 127.9 (s), 128.0 (d), 128.2 (d), 129.3 (d), 129.4 (d), 129.6 (s), 129.8 (s), 130.9 (d), 139.9 (s), 141.6 (s), 143.9 (s), 147.7 ppm (s) [16]. MS (EI, 70 eV): *m/z* (%) = 384 (61) [M]^+^, 305 (100), 231 (23). C_29_H_20_O (384.47): calcd. C 90.60, H 5.24; found C 90.32, H 5.19.

#### 6-(Biphenyl-2-yl)-7*H*-benzo[*de*]anthracen-7-one (**7**), 4,5-Dihydrospiro[benzo[*de*]anthracene-6,9′-fluoren]-7-ol (**11**), Bicyclo[4.3.1]decane derivative **12**, and 6-(Biphenyl-2-yl)-5,6-dihydro-4*H*-benzo[*de*]anthracen-7-ol (**13**)

The enol **4** (500 mg, 1.30 mmol) was dissolved in warm toluene (20 mL). Phosphoric acid (0.5 mL) and silica gel (1.0 g) were added and the mixture was stirred vigorously under reflux for 1 d. The solvent was removed and the crude product was fractioned by flash chromatography (CH_2_Cl_2_/*n*-hexane, 1:1, v/v; *R*_f_ = 0.73, 0.62, 0.51, 0.36). **First fraction:** 57 mg (11%) of **11** as a colourless microcrystalline solid with mp 228 °C (single crystals were grown from CDCl_3_). IR: 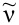  = 3473 (m), 3060 (w), 3033 (w), 2924 (w), 2892 (w), 2849 (w), 1592 (m), 1493 (w), 1437 (m), 1404 (m), 1286 (w), 1239 (w), 1208 (m), 1184 (w), 1160 (m), 1096 (w), 1077 (w), 964 (m), 925 (m), 894 (w), 808 (w), 752 (vs), 734 (s), 679 (m), 572 (m), 553 (m), 537 (m) cm^−1^. UV (acetonitrile): λ_max_ (log ε) = 196 (4.70), 210 (4.77), 260 (4.70), 304 (4.08), 345 (3.17), 362 (3.18) nm. ^1^H NMR (600 MHz, CDCl_3_): δ = 2.16 (t, *J* = 6.2 Hz, 2 H, C*H*_2_), 3.42 (t, *J* = 6.2 Hz, 2 H, C*H*_2_), 4.59 (s, 1 H, OH), 7.27–7.32 (m, 4 H), 7.45–7.75 (m, 5 H), 7.62 (ddd, *J* = 7.7, 6.9, 1.4 Hz, 1 H), 7.93 (ddd, *J* = 7.7, 0.9, 0.9 Hz, 2 H), 8.05 (dd, *J* = 8.3, 1.0 Hz, 1 H), 8.64 (dd, *J* = 8.2, 1.5 Hz, 1 H), 8.68 (d, *J* = 8.4 Hz, 1 H) ppm. ^13^C NMR (151 MHz, CDCl_3_): δ = 28.4 (t), 37.3 (t), 53.4 (s), 112.5 (s), 121.0 (d), 121.3 (d), 122.4 (d), 122.9 (d), 123.5 (d), 125.0 (d), 125.9 (s), 126.4 (d), 126.7 (d), 126.9 (d), 127.0 (s), 128.3 (s), 128.4 (s), 130.0 (s), 130.8 (s), 133.9 (s), 138.9 (s), 146.4 (s), 150.4 (s) ppm. *m/z* (%) = 384 (100) [M]^+^, 307 (63). HRMS: calcd. for C_29_H_20_O 384.151415 [M]^+^; found 384.15170. C_29_H_20_O (384.47): calcd. C 90.60, H 5.24; found C 90.17, H 5.15. **Second Fraction:** 57 mg (11%) of **13** as a colourless solid with mp 210 °C (single crystals were grown from d_6_-DMSO). IR: 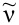  = 3554 (w), 3061 (w), 3021 (w), 2939 (w), 2921 (w), 2856 (w), 1599 (w), 1474 (w), 1460 (w), 1440 (w), 1414 (w), 1322 (w), 1267 (w), 1208 (m), 1162 (w), 1009 (w), 925 (w), 754 (vs), 738 (s), 706 (s), 663 (w), 616 (w), 597 (w), 545 (w) cm^−1^. UV (acetonitrile): λ_max_ (log ε) = 195 (4.83), 259 (4.65), 307 (3.99), 347 (3.11), 363 (3.11) nm. ^1^H NMR (400 MHz, d_6_-DMSO): δ = 1.68–1.77 (m, 2 H), 2.79–2.84 (m, 2 H), 5.27–5.32 (m, 1 H), 6.47 (d, *J* = 7.7 Hz, 1 H), 6.97–7.03 (m, 1 H), 7.15–7.20 (m, 2 H), 7.38 (dd, *J* = 7.0, 0.9 Hz, 1 H), 7.41–7.47 (m, 2 H), 7.55 (dd, *J* = 7.4, 7.4 Hz, 2 H), 7.61–7.72 (m, 4 H), 8.23–8.30 (m, 1 H), 8.66 (d, *J* = 7.7 Hz, 1 H), 8.84 (dd, *J* = 7.4, 2.2 Hz, 1 H), 9.27 (s, 1 H, O*H*) ppm. ^13^C NMR (101 MHz, d_6_-DMSO): δ = 25.0 (t), 27.2 (t), 34.7 (d), 117.9 (s), 121.0 (d), 122.2 (d), 123.2 (d), 123.4 (d), 125.7 (d), 125.7 (s), 126.2 (d), 126.3 (d), 126.3 (d), 126.3 (s), 126.6 (d), 127.0 (d), 127.7 (d), 128.3 (d), 129.2 (d), 130.0 (s), 130.1 (s), 130.2 (d), 134.3 (s), 141.2 (s), 141.8 (s), 142.7 (s), 145.9 (s) ppm. MS (EI, 70 eV): *m/z* (%) = 386 (56) [M]^+^, 231 (100), 232 (68). C_29_H_22_O (386.48): calcd. C 90.12, H 5.74; found C 90.27, H 5.81. **Third Fraction:** 47 mg (9%) of **12** as a colourless solid with mp 221 °C. IR: 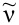  = 3563 (m), 3055 (w), 3022 (w), 2925 (w), 2863 (w), 1597 (w), 1492 (w), 1441 (w), 1422 (w), 1377 (w), 1324 (w), 1265 (w), 1226 (m), 1195 (w), 1160 (w), 974 (w), 961 (w), 946 (w), 839 (w), 764 (m), 746 (vs), 654 (w), 618 (w), 598 (m), 559 (m) cm^−1^. UV (acetonitrile): λ_max_ (log ε) = 215 (4.76), 259 (4.67), 316 (4.04), 349 (3.17), 366 (3.17) nm. ^1^H NMR (400 MHz, CDCl_3_): δ = 2.61 (ddd, *J* = 13.5, 2.8, 1.2 Hz, 1 H), 2.84 (ddd, *J* = 13.5, 7.0, 4.1 Hz, 1 H), 4.70–4.76 (m, 2 H), 6.05 (s, 1 H, O*H*), 7.03–7.11 (m, 2 H), 7.33–7.38 (m, 2 H), 7.39–7.47 (m, 3 H), 7.50–7.58 (m, 2 H), 7.60 (d, *J* = 6.9 Hz, 1 H), 7.63–7.66 (m, 1 H), 7.79–7.76 (m, 1 H), 8.17–8.20 (m, 1 H), 8.37 (dd, *J* = 8.4, 0.8 Hz, 1 H), 8.50–8.53 (m, 1 H) ppm. ^13^C NMR (101 MHz, CDCl_3_): δ = 31.3 (t), 41.3 (d), 46.2 (d), 114.8 (s), 120.7 (d), 121.9 (d), 122.6 (d), 123.9 (d), 124.3 (d), 126.0 (s), 126.2 (d), 126.3 (s), 126.4 (d), 126.6 (d), 127.1 (d), 127.8 (d), 127.9 (d), 128.4 (s), 128.5 (d), 130.5 (s), 131.6 (d), 131.8 (d), 134.1 (d), 136.1 (s), 138.7 (s), 140.9 (s), 141.1 (s), 144.3 (s), 147.6 (s) ppm. MS (EI, 70 eV): *m/z* (%) = 384 (100) [M]^+^, 231 (45). HRMS: calcd. for C_29_H_20_O 384.15142 [M]^+^; found 384.15113. C_29_H_20_O (384.47): calcd. C 90.60, H 5.24; found C 90.42, H 5.32. **Fourth Fraction:** 105 mg (21%) of **7** as a yellow solid with mp 197 °C (single crystals were grown from CH_2_Cl_2_/*n*-hexane, 1:1, v/v). IR: 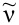  = 3056 (w), 3015 (w), 1645 (s), 1597 (m), 1558 (m), 1478 (m), 1463 (m), 1372 (w), 1349 (m), 1294 (m), 1264 (m), 1216 (w), 1173 (w), 1144 (w), 1072 (w), 1026 (w), 1007 (w), 939 (m), 919 (w), 898 (w), 846 (m), 828 (m), 780 (w), 750 (vs), 702 (s), 667 (m), 609 (w), 588 (m), 564 (w), 538 (m) cm^−1^. UV (acetonitrile): λ_max_ (log ε) = 205 (4.86), 232 (4.67), 256 (4.51), 362 (3.92), 385 (3.95) nm. ^1^H NMR (400 MHz, CDCl_3_): δ = 6.90–6.97 (m, 3 H), 7.11–7.14 (m, 2 H), 7.17–7.23 (m, 2 H), 7.36 (ddd, *J* = 7.4, 7,4, 1.6 Hz,1 H), 7.39–7.44 (m, 2 H), 7.47 (dd, *J* = 7.7, 1.6 Hz, 1 H), 7.58 (dd, *J* = 7.9, 7.5 Hz, 1 H), 7.62 (ddd, *J* = 7.5, 7.5, 1.5 Hz, 1 H), 7.84 (dd, *J* = 7.9, 0.8 Hz, 1 H), 7.86 (d, *J* = 8.4 Hz, 1 H), 8.22 (br. d, *J* = 8.0 Hz, 1 H), 8.23 (dd, *J* = 7.9, 1.5 Hz, 1 H), 8.39 (dd, *J* = 7.1, 0.8 Hz, 1 H) ppm. ^13^C NMR (101 MHz, CDCl_3_): δ = 122.6 (d), 124.2 (d), 126.2 (d), 126.4 (d), 126.5 (s), 127.3 (d), 127.4 (d), 127.7 (d), 128.1 (d), 128.1 (d), 128.5 (s), 128.9 (d), 129.5 (d), 130.1 (d), 130.1 (d), 131.6 (d), 132.1 (s), 132.2 (s), 132.9 (d), 133.3 (d), 135.5 (s), 139.5 (s), 141.5 (s), 142.4 (s), 146.8 (s), 184.2 (s) ppm [16]. MS (EI, 70 eV): *m/z* (%) = 382 (31) [M]^+^, 305 (100). C_29_H_18_O (382.45): calcd. C 91.07, H 4.74; found C 91.28, 4.74.

### X-Ray structure determinations

Numerical details are presented in Table 2. Data collection: Crystals were mounted in inert oil on glass fibres and transferred to the cold gas stream of the diffractometer (Oxford Diffraction Nova O for **7** and Bruker SMART 1000 CCDC for **11** and **13**). Crystals of compound **13** shattered at lower temperatures and were therefore measured at −90 °C. For **7** and **13**, an absorption correction based on multiple scans was performed. Structure refinement: The structures were refined anisotropically against *F*^2^ using the program SHELXL-97 [17]. The hydroxy hydrogens of **11** and **13** were refined freely; other H atoms were included using a riding model.

Complete crystallographic data (excluding structure factors) have been deposited at the Cambridge Crystallographic Data Centre under the numbers CCDC 705268 (**7**), 705269 (**11**) and 716351 (**13**).

**Table 2:** Details of X-ray structure analyses.

Compound	**7**	**11**	**13** × (CD_3_)_2_SO

Formula	C_29_H_18_O	C_29_H_20_O	C_31_H_22_D_6_O_2_S
*M*_r_	382.43	384.45	476.66
Habit	yellow tablet	colourless prism	colourless prism
Crystal size/mm	0.2 × 0.05 × 0.02	0.5 × 0.22 × 0.18	0.44 × 0.36 × 0.2
Radiation	Cu *K*α	Mo *K*α	Mo *K*α
λ/ Å	1.54184	0.71073	0.71073
Crystal system	monoclinic	monoclinic	orthorhombic
Space group	*P*2_1_/*c*	*P*2_1_/*c*	*Pccn*
Cell constants:			
*a*/Å	12.1176(4)	12.4948(8)	13.6065(4)
*b*/Å	18.8136(6)	13.8777(9)	19.9929(6)
*c*/Å	9.6914(4)	12.1737(8)	17.4749(6)
α/°	90	90	90
β/°	104.113(4)	115.461(4)	90
γ/°	90	90	90
*V*/Å^3^	1914.94	1905.9	4753.8
*Z*	4	4	8
*D*_x_/Mg m^−3^	1.327	1.340	1.332
μ/mm^−1^	0.61	0.08	0.16
*F*(000)	800	808	1968
*T*/°C	−170	−140	−90
2θ_max_	142	61	61
Completeness	97% to 135°	99.8% to 60°	99.1% to 60°
No. of reflections:			
measured	21722	29136	77542
independent	3534	5804	7094
*R*_int_	0.039	0.045	0.035
Parameters	271	275	313
*wR*(*F*^2^, all refl.)	0.107	0.128	0.094
*R*(*F*, >4σ(*F*))	0.041	0.044	0.037
*S*	1.02	1.03	0.92
max. Δρ/e Å^−3^	0.19	0.48	0.29

## Supporting Information

Supporting Information features copies of ^1^H and ^13^C NMR spectra of compounds **4**, **7**, **11**–**13**.

File 1 NMR spectra of compounds **4**, **7**, **11**–**13**.
