# Supplementary material for: A stable enol from a 6-substituted benzanthrone and its unexpected behaviour under acidic conditions
Source: Beilstein J Org Chem. 2009 Jun 16;5:31. doi: 10.3762/bjoc.5.31 (PMC2707016; doi:10.3762/bjoc.5.31)

Supporting Information

A stable enol from a 6-substituted benzanthrone and its unexpected behaviour under acidic conditions

Marc Debeaux[1], Kai Brandhorst[2], Peter G. Jones[3], Henning Hopf[2], Jörg Grunenberg[2], Wolfgang Kowalsky[1] and Hans-Hermann Johannes*[1]

[1] Labor für Elektrooptik am Institut für Hochfrequenztechnik, Technische Universität Braunschweig, Bienroder Weg 94, 38106 Braunschweig, Germany and

[2] Institut für Organische Chemie, Technische Universität Braunschweig, Hagenring 30, 38106 Braunschweig, Germany and

[3] Institut für Anorganische und Analytische Chemie, Technische Universität Braunschweig, Hagenring 30, 38106 Braunschweig, Germany

Email: Hans-Hermann Johannes – h2.johannes@ihf.tu-bs.de

* Corresponding author

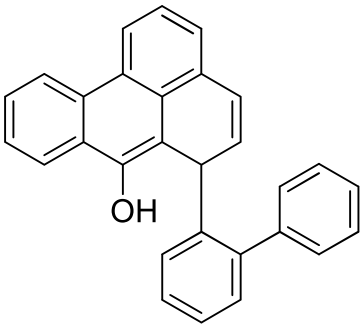

4

^{1}H NMR (400 MHz, d_6–DMSO):

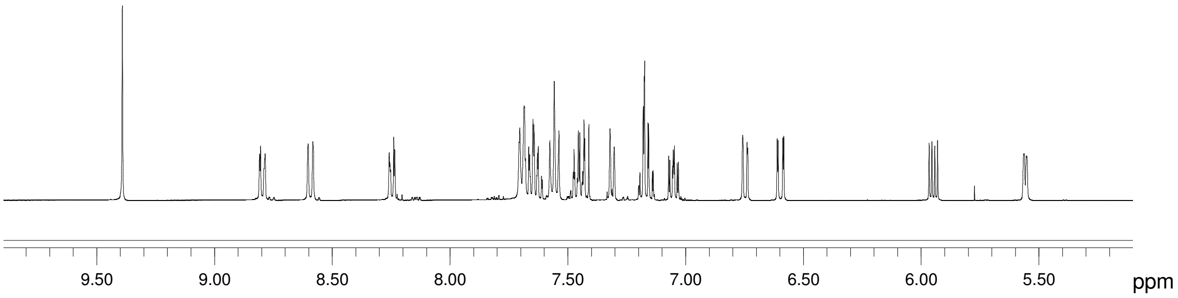

^{13}C NMR (101 MHz, d_6–DMSO):

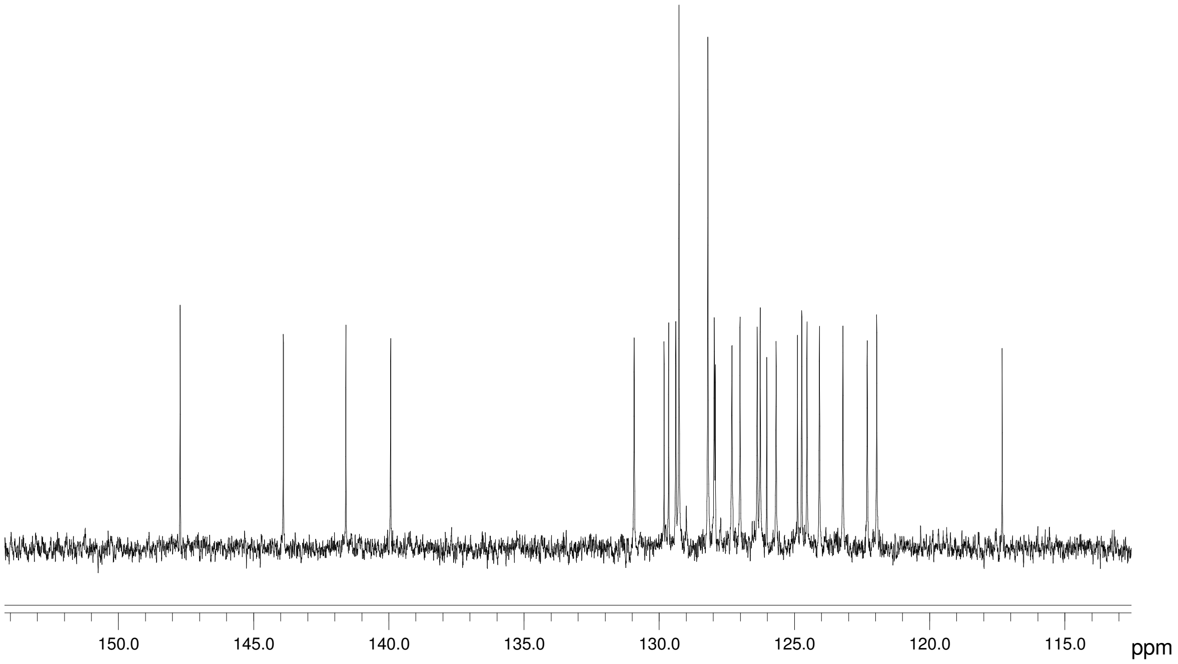

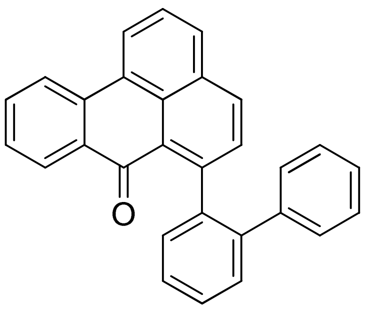

7

^{1}H NMR (400 MHz, $CDCl_3$):

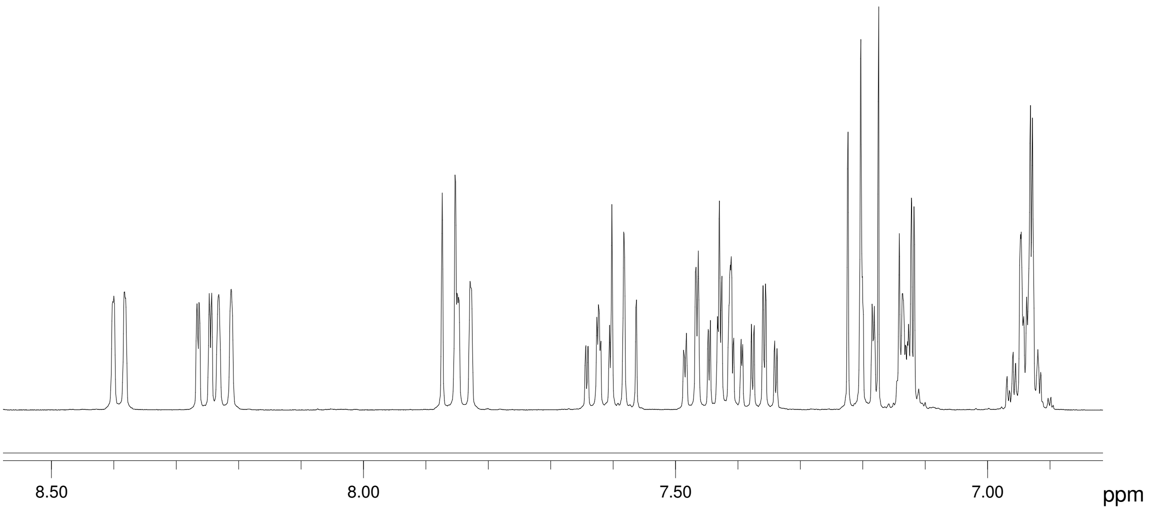

^{13}C NMR (101 MHz, $CDCl_3$):

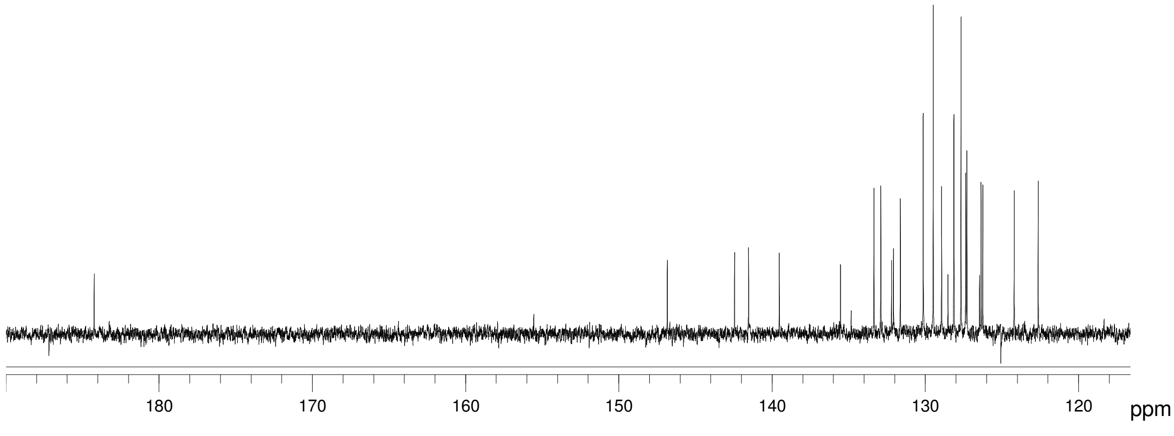

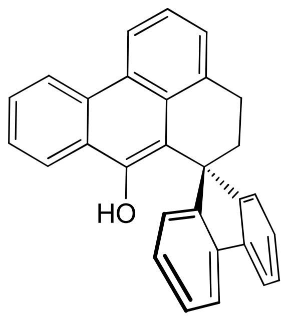

10

^{1}H NMR (600 MHz, $CDCl_3$):

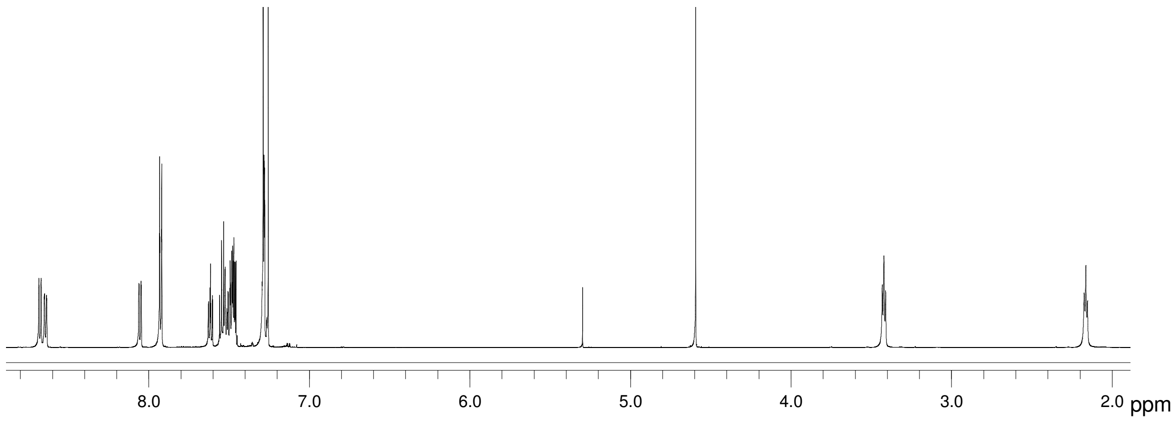

^{13}C NMR (151 MHz, $CDCl_3$):

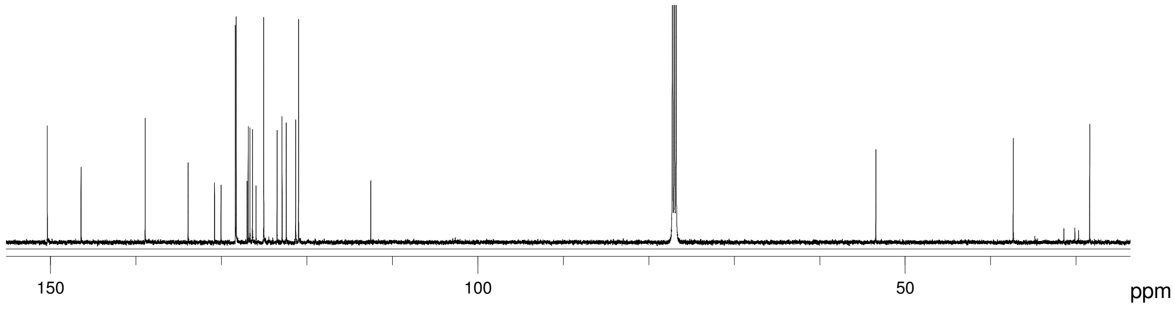

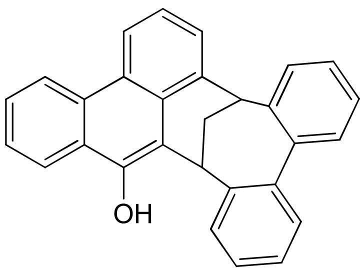

11

^{1}H NMR (400 MHz, CDCl$_3$):

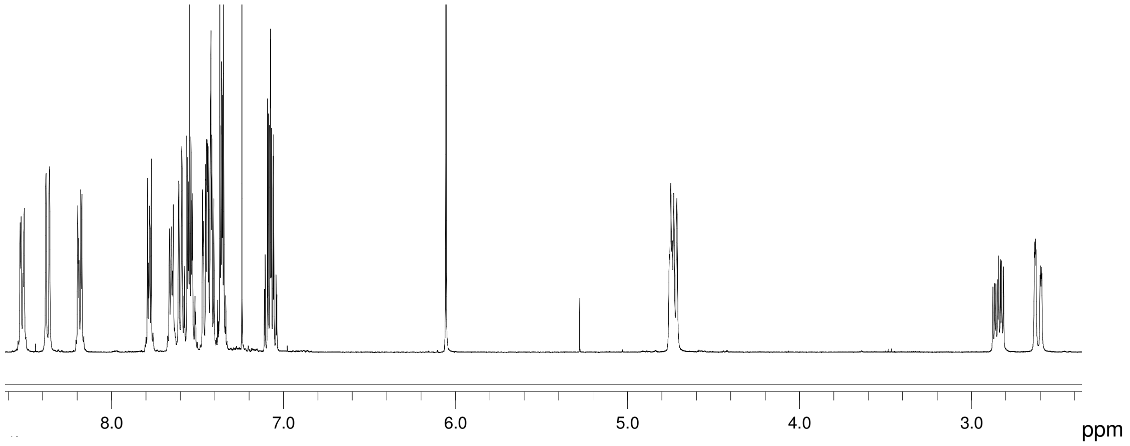

^{13}C NMR (101 MHz, CDCl$_3$):

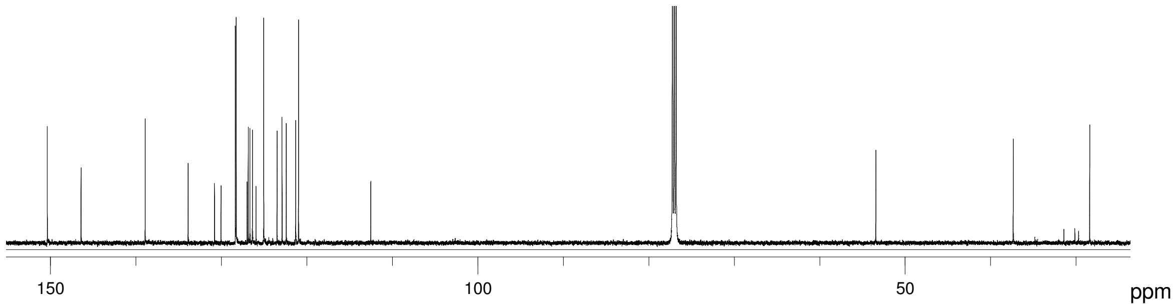

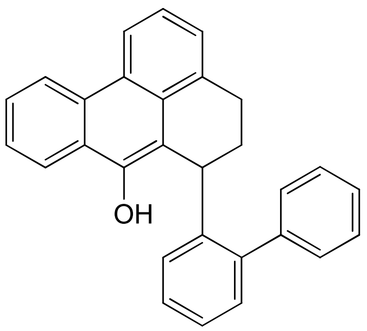

12

^{1}H NMR (400 MHz, d_6–DMSO):

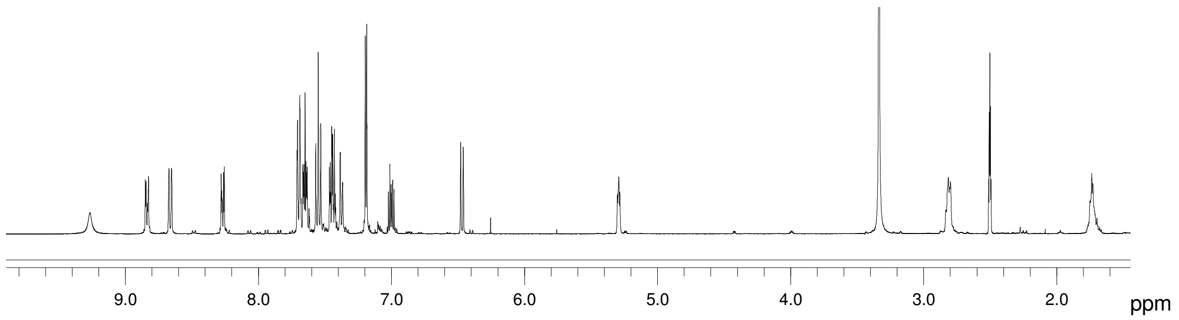

^{13}C NMR (101 MHz, d_6–DMSO):

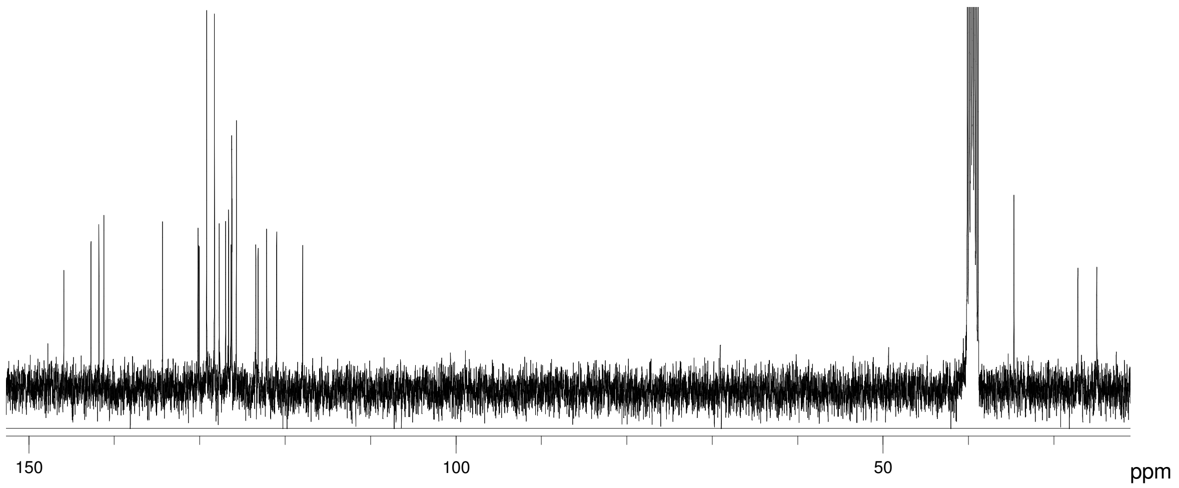

Supplement: File 1 — NMR spectra of compounds 4, 7, 11–13. [file Beilstein_J_Org_Chem-05-31-s001.pdf]
